# Effects of *Enterococcus faecalis* Supplementation on Growth Performance, Hepatic Lipid Metabolism, and mRNA Expression of Lipid Metabolism Genes and Intestinal Flora in Geese

**DOI:** 10.3390/ani15020268

**Published:** 2025-01-18

**Authors:** Siyu Sun, Yujie Zhao, Zhen Pang, Baoxia Wan, Jiaqi Wang, Zhenyu Wu, Qiuju Wang

**Affiliations:** Heilongjiang Provinal Key Laboratory of Exploration and Innovative Utilization of White Goose Germplasm Resources in Cold Region, College of Animal Science and Veterinary Medicine, Heilongjiang Bayi Agricultural University, Daqing 163319, China; ssy_166@163.com (S.S.); zyj2459964425@163.com (Y.Z.); 13614602377@163.com (Z.P.); 18746610692@163.com (B.W.); 13555043490@163.com (J.W.); 15847294490@163.com (Z.W.)

**Keywords:** goose, *E. faecalis*, liver lipid metabolism, gene expression, intestinal flora

## Abstract

The effects of *Enterococcus faecalis* (*E. faecalis*) at a concentration of 1.0 × 10^8^ CFU/mL on growth performance, hepatic lipid metabolism, and mRNA expression related to lipid metabolism, intestinal morphology, and intestinal flora were investigated in geese. A total of 60 male geese, aged 30 days and of similar weight, were randomly assigned to 2 groups. Each group was divided into six replicates, with five geese per replicate. During the 45-day experiment, the control group received a basal diet, while the experimental group was provided with the same basal diet supplemented with *E. faecalis* in drinking water at a concentration of 1.0 × 10^8^ CFU/mL. *E. faecalis* significantly increased the half-eviscerated weight of geese and improved ileal intestinal morphology (*p* < 0.05). Serum triglyceride (TG) levels were significantly reduced on day 5, while serum total cholesterol (TC) and low-density lipoprotein cholesterol (LDL-C) levels were significantly decreased on day 25 (*p* < 0.05). By day 45, serum TG and free fatty acid (FFA) levels were also significantly reduced (*p* < 0.05). Additionally, *E. faecalis* significantly increased the HDL/LDL ratio and serum high-density lipoprotein cholesterol (HDL-C) levels (*p* < 0.05). Serum insulin levels were significantly elevated on day 25, and glucagon-like peptide-1 (GLP-1) levels were significantly increased on day 45 (*p* < 0.05). On day 25 of the trial, hepatic TG levels, FFA levels, and Oil Red O-stained areas in the liver were significantly reduced (*p* < 0.05), accompanied by significantly decreased mRNA expression of hepatic acetyl-CoA carboxylase (ACCA) (*p* < 0.05). Conversely, the mRNA expression levels of fatty acid synthase (FASN), farnesoid X receptor (FXR), sterol regulatory element-binding protein 1 (SREBP-1), and peroxisome proliferator-activated receptor-α (PPARα) were significantly elevated (*p* < 0.05). A 16S rRNA diversity analysis of ileal contents on day 25 revealed significant differences in intestinal flora composition between the control and *E. faecalis* groups. The 16S rRNA data demonstrated a strong correlation between microbial communities and lipid-related physiological and biochemical indicators (*p* < 0.05). In conclusion, *E. faecalis* supplementation promoted fatty acid oxidation, reduced blood lipid levels, alleviated hepatic lipid accumulation, and improved ileal morphology and intestinal flora diversity, thereby enhancing growth performance and lipid metabolism in geese. These findings suggest that *E. faecalis* is a promising probiotic candidate for development as a feed additive.

## Simple Summary

Since the ban on antibiotics, there has been a growing interest in identifying probiotic alternatives. *Enterococcus faecalis* (*E. faecalis*) has emerged as a highly promising candidate strain. Additionally, lipid metabolism plays a critical role in animal production, yet research on geese remains limited. In this study, supplementation of *E. faecalis* in the drinking water of geese led to increased body weight and half-eviscerated weight, as well as reduced abdominal fat weight and hepatic lipid droplet content. Furthermore, serum levels of total cholesterol, triglycerides, and free fatty acids were significantly decreased. *E. faecalis* also exerted significant effects on hepatic lipid metabolism-related genes and improved ileal morphology and ileal microbiota diversity. In conclusion, this study demonstrates the beneficial effects of *E. faecalis* on growth performance and lipid metabolism in geese, providing a theoretical foundation for its potential application as a probiotic.

## 1. Introduction

Poultry are highly susceptible to fat deposition under intensive farming conditions. Fat deposition in poultry primarily includes subcutaneous fat, intermuscular fat, and abdominal fat, with abdominal fat being one of the main by-products of slaughter and often considered a waste product [1]. The rate of abdominal fat deposition is strongly correlated with overall body fat deposition in poultry, making it a reliable selection index for assessing fat deposition. Excessive body fat deposition is detrimental to healthy poultry production [2]. In broiler production, the energy required to produce a unit of fat is three times greater than that needed for a unit of lean meat, significantly reducing feed efficiency [3]. Abdominal fat is redundant during slaughter and processing, and its disposal decreases slaughter efficiency and economic viability. A healthy digestive environment is essential for optimal poultry growth and production yield [4]. Maintaining a healthy digestive tract minimizes nutrient waste, enhances production efficiency, and improves nutrient digestion and assimilation [5], while also reducing harmful gas emissions associated with poultry production. Gut health is protected by multiple barriers, including mechanical, immune, and bacterial barriers [6]. The diversity of gut microbiota plays a critical role in maintaining gut health in poultry. Intestinal microbes are vital for immunological functions and form a significant component of the immune system [7]. Gut health is influenced by the complex interplay between host immunity, microbial factors, and environmental conditions. Alterations in gut microbial composition can compromise the intestinal mucosal barrier, weaken immunity, and negatively impact poultry health.

In intensive poultry production, there is a growing global policy trend toward banning antibiotic-based feed additives [8], making the maintenance of poultry digestive health a critical issue that requires urgent attention. Probiotics have emerged as a promising alternative to antibiotics [9,10]. *Enterococcus faecalis* (*E. faecalis*), a Gram-positive probiotic bacterium, belongs to the class of *lactic acid bacteria* and the *Streptococcaceae* family [11]. It is commonly found in the intestinal tracts of humans and animals, where it functions as a symbiotic *lactic acid bacterium* [12]. Studies suggest that *E. faecalis* and its metabolite myristoleic acid (MA) may help reduce obesity by activating brown adipose tissue (BAT) and promoting beige fat formation [13]. Additionally, *E. faecalis* was shown to enhance nutrient absorption, preserve and maintain the integrity of the intestinal epithelial barrier, regulate the balance of the intestinal micro-ecosystem, and boost natural immunity [14,15]. Despite its diverse biological functions, research on *E. faecalis* in geese remains limited. In this study, we investigated the effects of *E. faecalis* at a concentration of 1.0 × 10^8^ CFU/mL in drinking water on growth performance, hepatic lipid metabolism, and mRNA expression related to lipid metabolism, gut morphology, and intestinal flora in geese. The findings provide a foundation for further research and empirical evidence supporting the role of *E. faecalis* in regulating lipid metabolism and its potential application in geese production.

## 2. Materials and Methods

### 2.1. Experimental Material

*E. faecalis* was isolated from the intestinal tract of Heilongjiang white geese by the Key Laboratory of Exploration and Innovative Utilization of White Goose Germplasm Resources in the Cold Region, Heilongjiang Province. The strain was deposited in the China Microbiology Depository Center (Deposit No. 24881). *E. faecalis* bacteria were included at a concentration of 1.0 × 10^8^ CFU/mL in drinking water.

### 2.2. Experimental Design and Geese Management

A total of 60 healthy 30-day-old male Heilongjiang white geese (Daqing, China) were randomly assigned to 2 groups (*n* = 30 per group). Each group was further divided into six replicates, with five geese per replicate. The control group received a basal diet (Table 1) and ordinary drinking water, while the experimental group was provided with the same basal diet supplemented with drinking water containing *E. faecalis* at a concentration of 1.0 × 10^8^ CFU/mL. The experiment lasted for 45 days. Both the experimental and control groups were housed in pens measuring 20 m in length and 15 m in width, equipped with a floor-based feeding system and a windowless rearing area. During the trial, the average low and high temperatures were 18 °C and 26 °C, respectively, with a relative humidity of 63%. The geese were fed twice daily at 8:00 a.m. and 4:30 p.m., with ad libitum access to water. All geese were vaccinated against avian influenza, goose paramyxovirus, and avian cholera.

### 2.3. Measurements of Growth Performance and Sample Collection

Body weight (BW) was measured before 7:00 a.m. on days 1, 7, 14, 21, 28, 35, and 42 of the trial. The feed conversion ratio (FCR), average daily gain (ADG), and average daily feed intake (ADFI) were calculated, and daily feed consumption was recorded using an electronic balance with an accuracy of 0.01 g (Shanghai Liangping Instrument Co., Ltd., Shanghai, China). On the mornings of days 5, 25, and 45 of the experiment, blood samples were collected at 07:00 from the wing veins of five randomly selected geese per group. The blood samples were centrifuged to separate the serum, which was then stored at −80 °C for subsequent analysis. Following blood collection, the geese were promptly anesthetized and humanely euthanized in accordance with established guidelines. Samples of abdominal fat, liver, mid-ileum, and ileal contents were collected from five geese per group. The weights of abdominal fat, trachea, gullet, crop, intestines, spleen, pancreas, gallbladder, reproductive organs, and liver were measured, and the half-eviscerated weight was calculated as follows: half-eviscerated weight = slaughter weight − (trachea + gullet + crop + intestines + spleen + pancreas + gallbladder + reproductive organs). Liver tissue samples were used to analyze the mRNA expression of genes and indicators related to hepatic lipid metabolism, assess gut morphology, and perform high-throughput sequencing of ileal microbiota.

### 2.4. Assessment of Serum and Hepatic Lipid Metabolism-Related Parameters

The serum lipid metabolism indicators free fatty acids (FFAs), total cholesterol (TC), triglyceride (TG), glucagon-like peptide-1 (GLP-1), high-density lipoprotein cholesterol (HDL-C), low-density lipoprotein cholesterol (LDL-C), glucagon, insulin, and leptin were assayed using commercial kits following the manufacturer’s instructions (Shanghai Enzyme-Link Bio-Technology Co., Ltd., Shanghai, China and Suzhou Grace Bio-Technology Co., Ltd., Suzhou, China).

### 2.5. Expression of Genes Related to Hepatic Lipid Metabolism

The expression levels of liver acetyl-coA carboxylase (ACCA), sterol regulatory element-binding protein 1 (SREBP1), farnesoid X receptor (FXR), fatty acid synthase (FASN), and peroxisome proliferator-activated receptor-α (PPARα) were assessed using real-time fluorescence quantitative nucleic acid amplification detection technology (q-PCR). Each group consisted of 5 replicates. For each replicate, approximately 1 g of liver tissue, previously stored at −80 °C, was weighed and homogenized in 1 mL of Trizol (Hunan Invitrogen Bio-Technology Co., Ltd., Hunan, China) using a ball mill (Verder Shanghai Instruments and Equipment Co., Ltd., Shanghai, China) until no solid residues remained. The homogenate was incubated at room temperature for 5 min, after which 200 μL of chloroform (Tianjin Kemiou Chemical Reagent Co., Ltd., Tianjin, China) was added, and the mixture was further incubated at room temperature for 10 min. The sample was then centrifuged at 12,000 rpm for 12 min at 4 °C using a refrigerated centrifuge (Model 2-16K, USA Sigma Co., Ltd., Burbank, CA, USA). Following centrifugation, 200 μL of the supernatant was transferred to a new EP tube, mixed with an equal volume of isopropanol, and incubated at room temperature for 10 min. The mixture was centrifuged again at 12,000 rpm for 12 min at 4 °C, and the supernatant was discarded. The pellet was washed twice with 75% ethanol, with each wash followed by centrifugation at 12,000 rpm for 7 min at 4 °C. After the second wash, the supernatant was discarded, and the pellet was air-dried on filter paper. Subsequently, 30 μL of DEPC water was added to dissolve the RNA, and reverse transcription was immediately performed using the PrimeScript™ RT Reagent Kit with gDNA Eraser (Dalian TaKaRa Biotechnology Co., Ltd., Dalian, China) on a PCR instrument (Model GE9612T-S, Hangzhou Bio-Gener Technology Co., Ltd., Hangzhou, China). The reverse transcription program was set as follows: 37 °C for 15 min, 50 °C for 5 min, and 85 °C for 5 s. After reverse transcription, 2 μL of cDNA was mixed with 10 μL of SYBR Green Premix Ex Taq II (Hunan Invitrogen Bio-Technology Co., Ltd.), 1 μL of forward primer, 1 μL of reverse primer, and 6 μL of DEPC water to prepare a 20 μL reaction system. The mixture was then subjected to quantitative PCR amplification using a fluorescence quantitative PCR instrument (JM1098, Bio-Rad Laboratories, Inc., Hercules, CA, USA). The conditions for amplification were as follows: pre-denaturation at 95 °C for 3 min, 40 cycles of 95 °C for 10 s, 56 °C for 30 s, 72 °C for 45 s, and finally 60 °C for 15 s. The amplification and lysis curves were observed.

The primers used for q-PCR are listed in Table 2, which were synthesized by Sangon Biotech (https://www.sangon.com) (accessed on 10 December 2023), and the data were analyzed via the 2^−ΔΔCT^ relative quantification method.

### 2.6. Histomorphological Observations

On days 5, 25, and 45 of the trial, frozen ileum sections were thawed, dried, and fixed in 4% paraformaldehyde for 15 min. The sections were then dehydrated in 75% alcohol, stained with hematoxylin for 3–5 min, and decolorized using hydrochloric acid. After rapid differentiation, the sections were exposed to ammonia to achieve a blue coloration. Intestinal tissue slices were examined and photographed under a Leica light microscope (DMILLED; Wetzlar, Germany) at a magnification of 10 × 20. Villus length, crypt depth, and the villus length-to-crypt depth ratio were analyzed using CaseViewer 2.4 software.

Frozen liver sections collected on days 5, 25, and 45 were fixed in 4% paraformaldehyde and stained with an Oil Red O working solution. After 8–10 min of staining (protected from light), background differentiation was performed. The sections were briefly excised and maintained for 3 s, followed by immersion in 60% isopropanol for 10 min. Nuclei were stained with hematoxylin for 3–5 min, and the sections were then immersed in a re-bluing solution for 1 s before being rinsed with tap water. Liver sections were examined and photographed under a Leica light microscope (DMILLED; Germany) at a magnification of 10 × 20. The stained areas were quantified using ImageJ 1.45 software.

### 2.7. Ileal Gut Microbiota Analysis via High-Throughput Sequencing

Genomic DNA was extracted from ileum samples using the CTAB/SDS method. The concentration and purity of the DNA were verified using a 1% agarose gel and a NanoDrop 2000 UV-Vis spectrophotometer (Thermo Fisher Scientific Co., Ltd., Waltham, MA, USA) The DNA was then diluted with ultrapure water to a final concentration of 1 µg/µL. The V3 + V4 region of the bacterial 16S rRNA gene was amplified using the primers 341F (5′-ACTCCTACGGGAGGCAGCA-3′) and 806R (5′-GGACTACHVGGGTWTCTAAT-3′). All libraries were constructed using the TruSeq^®^ DNA PCR-Free Sample Preparation Kit (Illumina, Inc., San Diego, CA, USA) and quantified using Qubit. Paired-end sequencing of community DNA fragments was performed on the Illumina platform. Sequence denoising was conducted using the DADA2 pipeline to obtain amplicon sequence variants (ASVs), and the length distribution of high-quality sequences was statistically analyzed. Taxonomic classification was performed using the QIIME2 (v.2019.4) classification sklarn algorithm with reference to the Greengenes, Silva, and Unite databases. Species annotation was carried out using a pre-trained Naive Bayes classifier, with default parameters in QIIME2 applied to each ASV or representative sequence of each operational taxonomic unit (OTU). Correlations between ileal microbiota and lipid metabolism metrics were analyzed using the R program (version 4.3.3) with the RStudio software, and the results were visualized as Spearman correlation heatmaps.

### 2.8. Statistical Analysis

Data were analyzed using SPSS 27.0 and GraphPad Prism 10.00. Results are presented as means ± standard error of the mean (SEM). Each experiment was independently repeated at least three times. Statistical significance was determined using two-tailed t-tests, with * *p* < 0.05 considered statistically significant, ** *p* < 0.01 indicating high significance, and *** *p* < 0.001 representing extreme significance.

## 3. Results

### 3.1. Growth Performance

The addition of *E. faecalis* to the drinking water of geese significantly affected several growth performance parameters, including FCR, ADG, BW, and ADFI (Table 3). No significant differences in initial body weight were observed among the groups. Compared to the control group, geese in the *E. faecalis* group exhibited a significant reduction in body weight on day 5 (*p* < 0.05), followed by a significant increase on days 25 and 45 (*p* < 0.05). From day 1 to day 5, the ADG in the *E. faecalis* group was significantly lower than that in the CON group (*p* < 0.05). However, during the periods of 5–25 days and 1–45 days, the ADG in the *E. faecalis* group was significantly higher compared to the CON group (*p* < 0.05). Compared to the CON group, the ADFI in the *E. faecalis* group was significantly lower during the periods of 5–25 days, 25–45 days, and 1–45 days (*p* < 0.05). Additionally, the FCR in the *E. faecalis* group was significantly lower during the 1–45 day period compared to the CON group (*p* < 0.05).

### 3.2. Slaughtering Indicators

Table 4 summarizes the effects of *E. faecalis* supplementation in drinking water on abdominal fat weight, liver weight, and half-eviscerated weight in geese. Compared to the CON group, the abdominal fat weight and liver weight in the *E. faecalis* group were significantly lower on days 5 and 45 (*p* < 0.05). On day 5, the half-eviscerated weight in the *E. faecalis* group was significantly lower than that in the CON group (*p* < 0.05). In contrast, on days 25 and 45, the half-eviscerated weight in the *E. faecalis* group was significantly higher compared to the CON group (*p* < 0.05).

### 3.3. Serum Indicators of Lipid Metabolism

**Figure 1:**
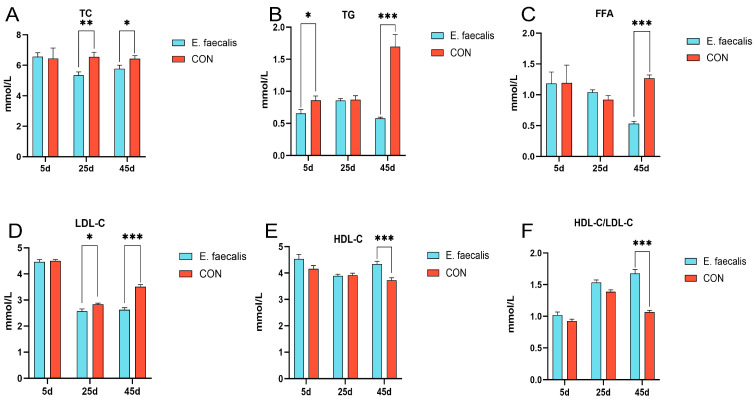
Effect of *Enterococcus faecalis* on indices of serum lipid metabolism in geese. (**A**) Total cholesterol (TC) concentration in serum. (**B**) Serum triglyceride (TG) concentration. (**C**) Serum free fatty acid (FFA) content. (**D**) Serum high-density lipoprotein cholesterol (HDL-C) content. (**E**) Serum low-density lipoprotein cholesterol (LDL-C) level. (**F**) Serum high-density lipoprotein cholesterol/low-density lipoprotein cholesterol (HDL-C/LDL-C) content. CON = control; *E. faecalis* = drinking water with an *Enterococcus faecalis* concentration of 1 × 10^8^ CFU/mL. * *p* < 0.05, ** *p* < 0.01, and *** *p* < 0.001 compared to CON.

Figure 1A–F illustrates the levels of FFA, HDL-C, TC, TG, LDL-C, and HDL-C/LDL-C in the serum samples collected on days 5, 25, and 45. These parameters were analyzed to assess the effects of *E. faecalis* supplementation on serum lipid metabolism in geese. Compared to the CON group, the serum TC level in the *E. faecalis* group was significantly lower on day 25 (*p* < 0.01) and significantly lower on day 45 (*p* < 0.05) (Figure 1A). The serum TG levels in the *E. faecalis* group were significantly lower than those in the CON group on day 5 (*p* < 0.05) and extremely significantly lower on day 45 (*p* < 0.001) (Figure 1B). On day 45, both FFA and LDL-C levels in the *E. faecalis* group were extremely significantly lower than those in the CON group (*p* < 0.001) (Figure 1C,E). Additionally, on day 25, the LDL-C levels in the *E. faecalis* group were significantly lower than those in the CON group (*p* < 0.05). By day 45, the HDL-C and HDL-C/LDL-C levels in the *E. faecalis* group were extremely significantly higher than those in the CON group (*p* < 0.001) (Figure 1D,F).

### 3.4. Serum Lipid Metabolism-Related Hormone Indicators

**Figure 2:**
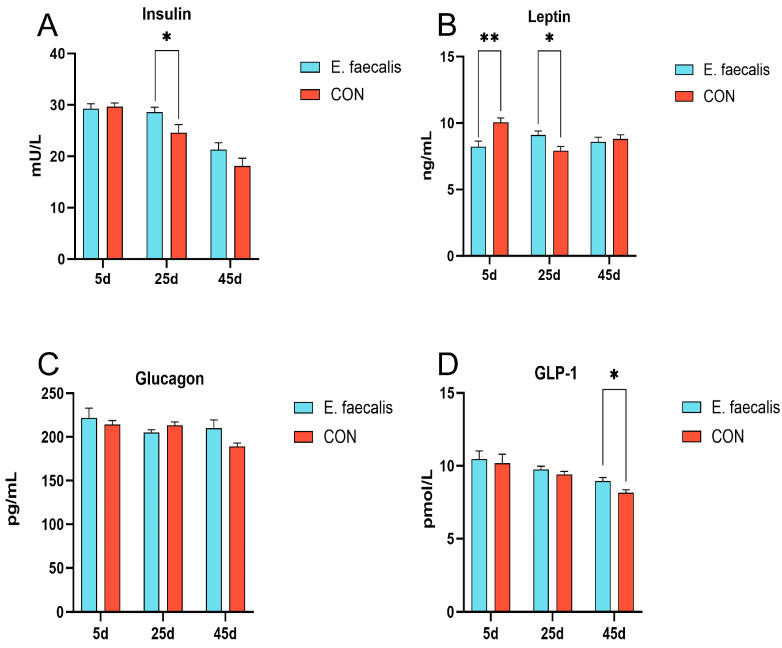
Effect of *Enterococcus faecalis* on indices of serum hormone metabolism in geese. (**A**) Insulin (insulin) content. (**B**) Leptin content. (**C**) Glucagon content. (**D**) Serum glucagon-like peptide 1 (GLP-1) content. CON = control; *E. faecalis* = drinking water with an *Enterococcus faecalis* concentration of 1.0 × 10^8^ CFU/mL. * *p* < 0.05, ** *p* < 0.01 compared to CON.

The effects of *E. faecalis* on serum hormone metabolic indices in geese are presented in Figure 2. The levels of insulin, leptin, glucagon, and GLP-1 in the serum samples collected on days 5, 25, and 45 were measured to evaluate these effects (Figure 2A–D). On day 25, the serum insulin level in the *E. faecalis* group was significantly higher than that in the CON group (*p* < 0.05) (Figure 2A). Similarly, on day 5, the serum leptin level in the *E. faecalis* group was significantly higher than that in the CON group (*p* < 0.01). By day 25, the serum leptin level in the *E. faecalis* group remained significantly higher compared to the CON group (*p* < 0.05) (Figure 2B). In contrast, no significant difference in serum glucagon levels was observed between the *E. faecalis* and CON groups (*p* > 0.05) (Figure 2C). Additionally, no statistically significant difference in serum GLP-1 levels was observed between the *E. faecalis* and CON groups on days 5 and 25. However, on day 45, the serum GLP-1 level in the *E. faecalis* group was significantly higher than that in the CON group (*p* < 0.05) (Figure 2D).

### 3.5. Indicators of Hepatic Lipid Metabolism

**Figure 3:**
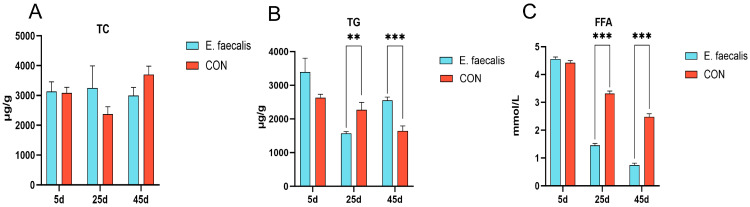
Impact of *Enterococcus faecalis* on lipid metabolism parameters in goose liver. (**A**) Liver total cholesterol (TC) content. (**B**) Hepatic triglyceride (TG) content. (**C**) Liver free fatty acid (FFA) content. CON = control group; *E. faecalis* = drinking water with an *Enterococcus faecalis* concentration of 1.0 × 10^8^ CFU/mL. ** *p* < 0.01 and *** *p* < 0.001 compared to CON.

Figure 3 illustrates the effects of *E. faecalis* on hepatic lipid metabolism parameters in geese. The levels of TC, TG, and FFA in the liver samples collected on days 5, 25, and 45 were measured to evaluate the impact of *E. faecalis* supplementation on lipid metabolism-related indices (Figure 3A–C). No significant difference in hepatic TC levels was observed between the *E. faecalis* and CON groups (*p* > 0.05) (Figure 3A). In contrast, the hepatic TG level in the *E. faecalis* group was significantly lower than that in the CON group on day 25 (*p* < 0.01). Conversely, on day 45, the hepatic TG level in the *E. faecalis* group was extremely significantly higher compared to the CON group (*p* < 0.001) (Figure 3B). Similarly, the hepatic FFA levels in the *E. faecalis* group were extremely significantly lower than those in the CON group on days 25 and 45 (*p* < 0.001) (Figure 3C).

### 3.6. Gene Expression Related to Hepatic Lipid Metabolism

**Figure 4:**
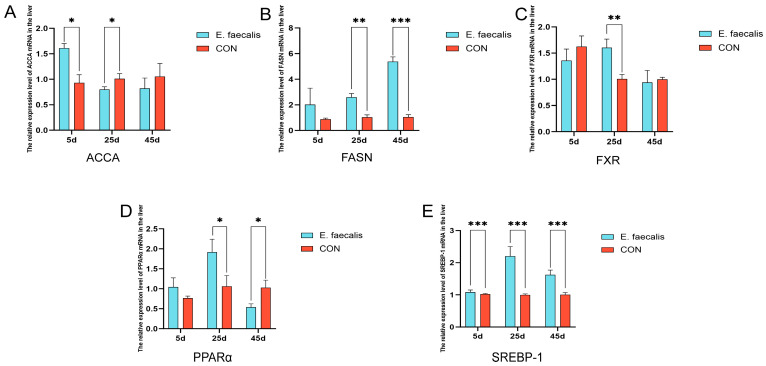
Effect of *Enterococcus faecalis* on mRNA expression of genes related to lipid metabolism in goose liver. (**A**) Liver *ACCA* mRNA expression levels. (**B**) Liver *FASN* mRNA expression levels. (**C**) Hepatic *FXR* mRNA expression level. (**D**) Hepatic *PPARα* mRNA expression level. (**E**) Liver *SREBP-1* mRNA expression level. CON = control group; *E. faecalis* = drinking water with an *Enterococcus faecalis* concentration of 1.0 × 10^8^ CFU/mL. * *p* < 0.05, ** *p* < 0.01, and *** *p* < 0.001 compared to CON.

Figure 4 illustrates the impact of *E. faecalis* on the expression of genes associated with hepatic lipid metabolism. The mRNA levels of key lipid metabolism-related genes, including *ACCA*, *FASN*, *FXR*, *PPARα*, and *SREBP-1*, were quantified in liver samples collected on days 5, 25, and 45 of the study (Figure 4A–E). On day 5, the mRNA expression of *ACCA* in the *E. faecalis* group was significantly higher than that in the CON group (*p* < 0.05). In contrast, by day 25, *ACCA* mRNA expression in the *E. faecalis* group was significantly lower compared to the CON group (*p* < 0.05). Similarly, the mRNA expression of *FASN* in the *E. faecalis* group was significantly higher than that in the CON group on day 25 (*p* < 0.01) and extremely significantly higher on day 45 (*p* < 0.001) (Figure 4B). On day 25, the mRNA expression of *FXR* in the *E. faecalis* group was significantly higher than that in the CON group (*p* < 0.01) (Figure 4C). The mRNA expression of *PPARα* in the *E. faecalis* group was significantly higher than that in the CON group on day 25 (*p* < 0.05). Conversely, on day 45, *PPARα* mRNA expression in the *E. faecalis* group was significantly lower compared to the CON group (*p* < 0.05) (Figure 4D). Furthermore, the mRNA expression of *SREBP-1* in the *E. faecalis* group was extremely significantly higher than that in the CON group on days 5, 25, and 45 (*p* < 0.001) (Figure 4E).

### 3.7. Ileum Morphology

**Figure 5:**
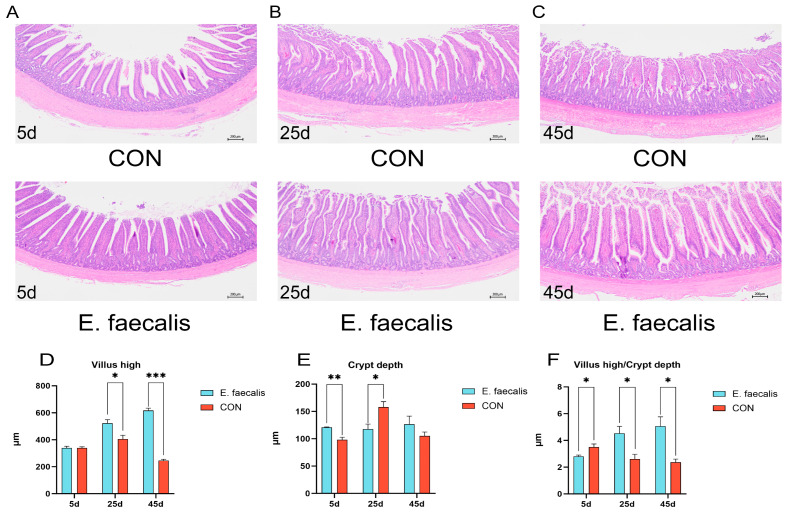
HE staining to analyze ileum morphology. (**A**) Ileal section on day 5 of experiment. (**B**) Ileal section on day 25 of test. (**C**) Ileal section on day 45 of experiment. (**D**) Height of villi. (**E**) Crypt depth. (**F**) Chorionic villus height/crypt depth. CON = control group; *E. faecalis* = drinking water with an *Enterococcus faecalis* concentration of 1.0 × 10^8^ CFU/mL. * *p* < 0.05, ** *p* < 0.01, and *** *p* < 0.001 compared to CON.

Figure 5A–C illustrates the ileal morphology following hematoxylin and eosin (HE) staining on days 5, 25, and 45 of the trial. In the *E. faecalis* group, the heights of intestinal villi were significantly greater than those in the CON group on days 25 and 45 (*p* < 0.05; *p* < 0.001). On day 5, the *E. faecalis* group exhibited significantly deeper crypt depths compared to the CON group (*p* < 0.01), while on day 25, crypt depths were significantly reduced (*p* < 0.05). The ratio of ileal villus height to crypt depth was significantly lower in the *E. faecalis* group compared to the CON group on day 5 (*p* < 0.05). In contrast, on days 25 and 45, the *E. faecalis* group demonstrated significantly higher ratios of villus height to crypt depth than the CON group (*p* < 0.05) (Figure 5F).

### 3.8. Lipid Droplet Deposition in Liver

**Figure 6:**
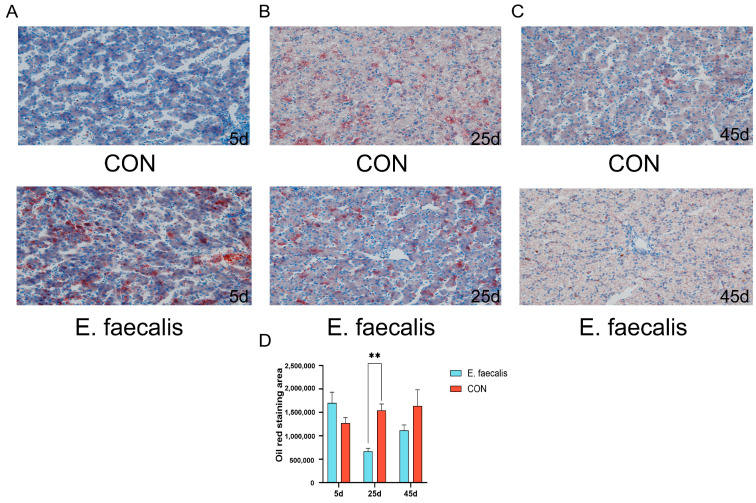
Oil Red O staining is utilized to assess the extent of hepatic lipid droplets. (**A**) Sections on day 5 of the experiment. (**B**) Sections on day 25 of the experiment. (**C**) Sections on day 45 of the experiment. (**D**) The Oil Red stained area. CON = control group; *E. faecalis* = drinking water with an *Enterococcus faecalis* concentration of 1.0 × 10^8^ CFU/mL. ** *p* < 0.01 compared to CON.

Figure 6A–C presents the results of Oil Red O staining of the liver tissues on days 5, 25, and 45 of the experiment. On day 25, the *E. faecalis* group exhibited significantly reduced Oil Red O staining in the liver compared to the CON group (*p* < 0.05; Figure 6D).

### 3.9. Ileal Intestinal Flora Diversity Analysis

**Figure 7:**
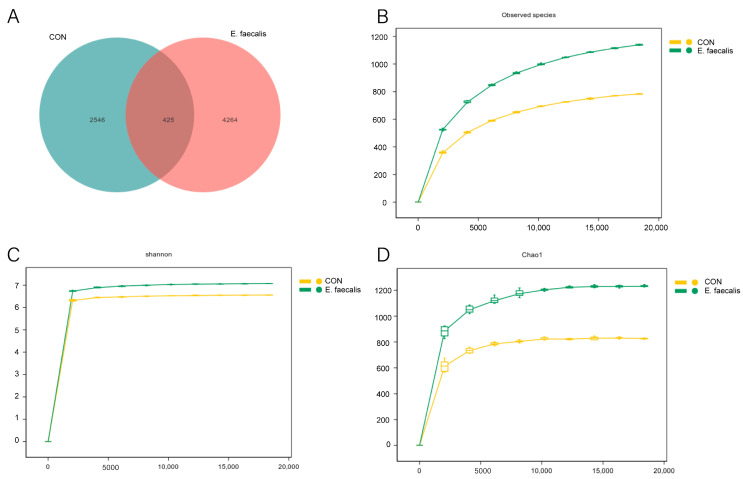
Evaluation of quality of ileal colony sequencing data and ASV quantifications. (**A**) Venn diagram on 25th day of trial. (**B**) Sparse curve of Observed_species on day 25 of trial. (**C**) Sparse curve of Shannon on day 25 of trial. (**D**) Sparse curve of Chao1 on day 25 of trial. CON = control group; *E. faecalis* = drinking water with an *Enterococcus faecalis* concentration of 1.0 × 10^8^ CFU/mL.

To investigate the effects of *E. faecalis* supplementation on the intestinal microbiota of geese and to integrate findings from pre-serum, liver, and ileum morphology analyses, amplicon sequencing was performed on ileal content samples collected on day 25 of the experiment. The CON group exhibited 2971 core amplicon sequence variants (ASVs), while the *E. faecalis* group had 4689 core ASVs, with 425 ASVs shared between the 2 groups. The sparse and sorted abundance curves (Figure 7B–D) confirmed adequate sequencing depth and uniformity.

### 3.10. Alpha and Beta Diversity Analyses

**Figure 8:**
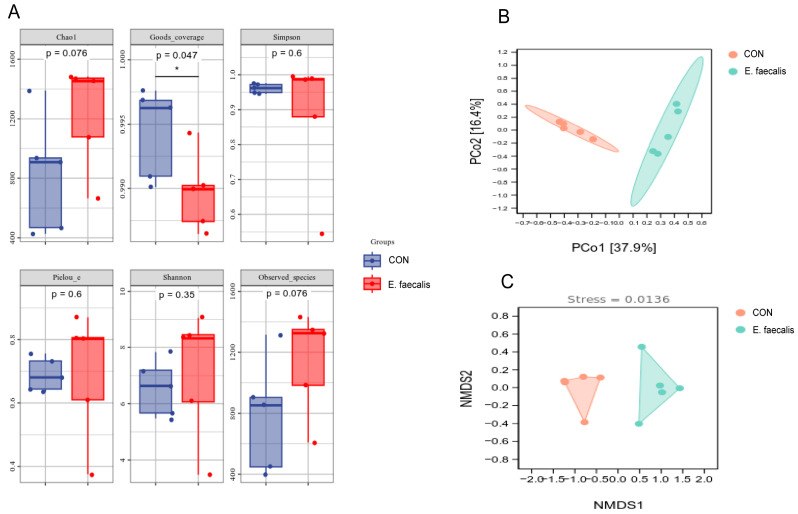
The alpha and beta diversity indices on day 25 of the experiment. (**A**) Alpha diversity on day 25 of the experiment. (**B**) The principal coordinate analysis (PCoA) of the bacterial communities on trial day 25 using bray_curtis distances. (**C**) NMDS-based Beta diversity analysis on day 5 of the trial. *E. faecalis* = drinking water with an *Enterococcus faecalis* concentration of 1.0 × 10^8^ CFU/mL.

We evaluated the effect of *E. faecalis* on the bacterial diversity of the ileum in geese by calculating several alpha diversity indices to analyze alterations in gut microbiota (Figure 8). The Goods coverage was used as an index to reflect the sequencing depth. The lowest index in this test was 98.65%, while the highest index was 99.76%. There was a significant difference between the two groups, indicating that the sequencing depth of this study was reasonable. The Chao1, Observed_species, Shannon, Simpson, and Pielou evenness indices all showed an upward trend in the *E. faecalis* group (Figure 8A). Thus, adding *E. faecalis* to drinking water may improve the quantity, variety, and uniformity of the gut flora.

The reduction in multidimensional species data to assess differences in the composition of bacterial communities between the two groups is shown in Figure 8B. A principal coordinate analysis (PCoA) was performed using the Bray–Curtis distance algorithm. The PCoA results showed a clear separation between samples belonging to the CON and *E. faecalis* groups. NMDS analysis, an essential metric of the sample variance, indicates that stress levels less than 0.2 denote significant variability across samples. The stress value of 0.0136 (<0.2) in Figure 8C indicates a substantial variation in community makeup between the CON and *E. faecalis* groups.

### 3.11. Analysis of Species Differences

**Figure 9:**
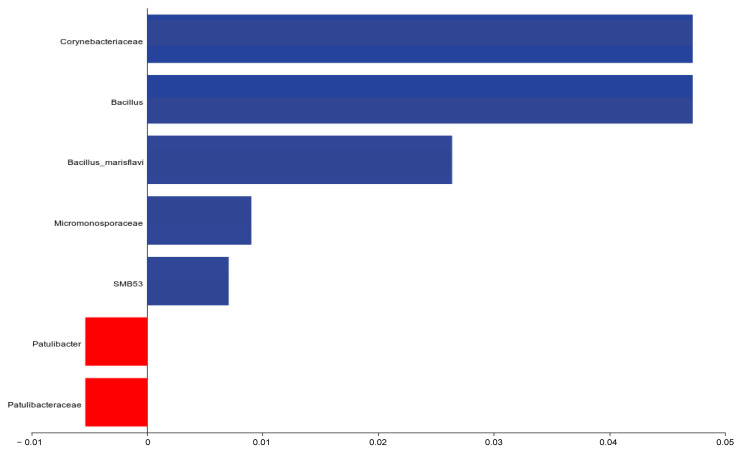
Differential ileal gut microbiota on day 25 of trial. CON = control group; *E. faecalis* = drinking water with an *Enterococcus faecalis* concentration of 1.0 × 10^8^ CFU/m.

An analysis of the differential bacterial composition (Figure 9) showed that *Corynebacteriaceae*, *Bacillus*, *Bacillus_marisflavi*, *Micromonosporaceae*, and *SMB53* in the *E. faecalis* group exhibited significant changes on day 25 of the experiment. Notable differences were identified within the CON group regarding *Patulibacteraceae* and *Patulibacter*.

### 3.12. Relationship Between Intestinal Microbiota and Lipid Metabolism Markers

**Figure 10:**
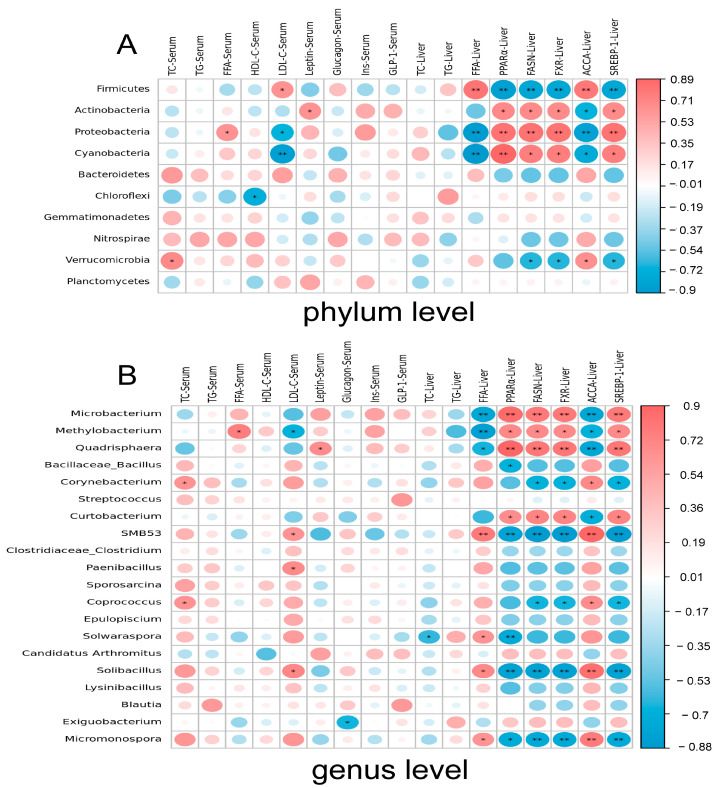
Spearman correlation analysis. (**A**) On trial day 25, Spearman’s correlation analysis of changes in serum lipid metabolism index, hepatic lipid metabolism index, hepatic lipid metabolism pathway genes, and intestinal microbiota (phylum level). (**B**) On trial day 25, Spearman correlation analysis of changes in serum lipid metabolism index, hepatic lipid metabolism index, hepatic lipid metabolism-related genes, and gut microbiota (genus level). * *p* < 0.05 and ** *p* < 0.01 compared to CON. Red represents positive correlation, blue represents negative correlation, and white represents no correlation.

The leading 10 bacteria at the gate level were associated with indicators of lipid metabolism (Figure 10A). *Firmicutes* were positively associated with serum LDL-C and liver FFA, and negatively correlated with *PPARα*, *FASN*, *FXR*, and *SREBP-1* on day 25 of the experiment. *Actinobacteria* were positively correlated with serum leptin, liver *PPARα*, liver *FASN*, liver *FXR*, and liver *SREBP-1* and negatively correlated with liver *ACCA*. Serum FFA, liver *PPARα*, liver *FASN*, liver *FXR*, and liver *SREBP-1* were positively correlated with *Proteobacteria*, while serum LDL-C, liver FFA, and liver *ACCA* were negatively correlated. *Cyanobacteria* were positively correlated with liver *PPARα*, liver *FASN*, liver *FXR*, and liver *SREBP-1* and negatively correlated with serum LDL-C, liver FFA, and liver *ACCA*. *Chloroflexi* was negatively correlated with serum HDL-C. *Verrucomicrobia* was positively correlated with serum TC and liver *ACCA* and negatively correlated with liver *FASN*, liver *FXR*, and liver *SREBP-1*.

The top 20 bacteria at the genus level were included in a correlation analysis with lipid metabolism indicators (Figure 10B). The analysis showed that *Microbacterium* was positively associated with liver *PPARα*, liver *FASN*, liver *FXR*, and liver *SREBP-1* and negatively correlated with liver FFA and liver *ACCA* on day 25 of the trial. *Methylobacterium* was positively correlated with serum FFA, liver *PPARα*, liver *FASN*, liver *FXR*, and liver *SREBP-1* and negatively correlated with liver FFA and liver *ACCA*. *Quarisphaera* was positively correlated with serum leptin, liver *PPARα*, liver *FASN*, liver *FXR*, and liver *SREBP-1* and negatively correlated with liver FFA and liver *ACCA*. *Bacillaceae-Bacillus* was negatively correlated with liver *PPARα*. *Corynebacterium* was positively correlated with serum TC and hepatic *ACCA* and negatively correlated with hepatic *FASN*, hepatic *FXR*, and liver *SREBP-1*. *Curtobacterium* was positively correlated with liver *PPARα*, liver *FASN*, liver *FXR*, and liver *SREBP-1* and negatively correlated with hepatic *ACCA*. *SMB53* showed a positive correlation with serum LDL-C, liver FFA, and liver *ACCA* but had a negative correlation with liver *PPARα*, liver *FASN*, liver *FXR*, and liver *SREBP-1*. *Paenibacillus* was positively correlated with serum LDL-C. *Coprococcus* was positively correlated with serum TC and liver *ACCA* and negatively correlated with liver *FASN*, liver *FXR,* and liver *SREBP-1*. *Solwaraspora* had a positive correlation with liver FFA and a negative correlation with liver TC and liver *PPARα*. *Solibacillus* had a positive correlation with serum LDL-C, liver FFA, and liver *ACCA* and a negative correlation with liver *PPARα*, liver *FASN*, liver *FXR*, and liver *SREBP-1*. Serum glucagon was negatively correlated with *Exiguobacterium*. *Micromonospora* had positive correlations with liver FFA and *ACCA* and negative correlations with *PPARα*, *FASN*, *FXR*, and *SREBP-1*.

## 4. Discussion

In this study, we evaluated the effects of probiotic *E. faecalis* on growth performance, serum and liver lipid metabolism, ileal morphology, and ileal microbiological composition of white geese in Heilongjiang Province. This strain has demonstrated beneficial effects as a component of mixed probiotics in broilers when used as a feed additive [16]. However, its efficacy in geese remains unknown. Therefore, it is necessary to conduct a study to investigate the effects of *E. faecalis* as a feed additive in geese.

BW gain is one of the critical indicators of organismal growth, reflecting nutrient absorption, metabolic efficiency, and overall health status. In the present study, as is consistent with previous findings, the supplementation of compound probiotics containing *E. faecalis* significantly increased BW and ADG in piglets, while significantly reducing FCR [17]. These results were in line with expectations. However, the significantly lower BW on day 5 and ADG from days 1 to 5 in the *E. faecalis* group compared to the CON group may be attributed to the initial colonization period of *E. faecalis* and the activation of the immune system. Nevertheless, as the colonization of *E. faecalis* was completed and intestinal function improved, the geese exhibited significant compensatory growth during the 35–75 day period, ultimately achieving superior growth performance compared to the CON group. The ADFI in the *E. faecalis* group was significantly lower than that in the CON group. Previous studies have demonstrated that gut microbiota plays a pivotal role in regulating appetite and satiety, influencing feed intake through various mechanisms, including the release of satiety peptides and the modulation of reward pathways [18], which may explain the reduced ADFI observed in the *E. faecalis* group in this study. Regarding FCR, the values in this study were relatively higher compared to other studies. However, it was reported that the growth curve of geese typically follows an “S” shape, with a significant slowdown in weight gain during the later stages of growth [19], which is considered a normal physiological phenomenon. In this study, the extended experimental duration resulted in slower weight gain during the later growth stages, leading to a higher FCR from days 1 to 45. This also explains why we selected geese at day 25 of the experiment to analyze the intestinal microbiota.

Unstable fat metabolism often leads to unnecessary fat deposition [20]. Compared to the control group, supplementation with *E. faecalis* significantly improved abdominal fat weight in geese, suggesting that *E. faecalis* may reduce abdominal fat deposition by modulating lipid metabolism mechanisms. This finding is consistent with previous studies demonstrating that probiotics can modulate lipid metabolism in broilers by reducing total cholesterol and triglyceride levels in ileal epithelial cells and upregulating the AMP-activated protein kinase alpha (AMPKα)/PPARα/Carnitine palmitoyltransferase 1 (CPT-1) pathway in the liver, thereby influencing abdominal fat deposition [21]. Additionally, the significant reduction in liver weight observed in this study, particularly a 33% decrease in the *E. faecalis* group on day 45, may be attributed to the anti-inflammatory effects of *E. faecalis*, which potentially alleviate hepatic inflammation and associated hypertrophy. Previous studies have demonstrated that probiotics can mitigate liver inflammation by increasing natural killer T (NKT) cells in the liver [22], while lactic acid bacteria reduce liver weight by improving the expression of inflammatory cytokines [23]. Based on these findings, we speculate that the significant reduction in liver weight observed in this study may be related to the regulation of hepatic lipid metabolism and the anti-inflammatory effects of *E. faecalis*. Half-eviscerated weight, a direct indicator of animal growth performance, reflects nutrient absorption and utilization efficiency during the rearing period. Higher carcass weight typically indicates improved slaughter yield and economic benefits. It was reported that probiotic supplementation increases carcass weight in male laying hens [21], and other studies have shown that both natural and commercial probiotics enhance carcass weight in broilers [24], which is consistent with the slaughter performance results observed in this study and aligns with expectations. In conclusion, supplementation with *E. faecalis* not only improved lipid metabolism and liver health in geese but also enhanced slaughter performance, demonstrating potential economic benefits.

Serum biochemical parameters are critical indicators of lipid metabolism. In the present study, supplementation with *E. faecalis* significantly reduced serum levels of TC, TG, FFA, and LDL-C, while significantly increasing HDL-C levels in geese. These findings suggest that *E. faecalis* effectively regulates lipid metabolism and reduces blood cholesterol levels in geese. Furthermore, previous studies have demonstrated that supplementation with lactic acid bacteria significantly reduces TC and LDL-C levels in the blood [25], which is consistent with the results of this study. Additionally, probiotics were shown to significantly decrease serum TG levels in rats [26], and both single-strain and multi-strain probiotics significantly reduce serum FFA levels in non-alcoholic steatohepatitis (NASH) rats [27]. Moreover, probiotic supplementation in hamsters fed a high-cholesterol diet (HCD) significantly increased serum HDL-C levels and the HDL-C/LDL-C ratio, while significantly reducing TC, TG, and FFA levels [28]. These results further confirm the important role of probiotics in modulating lipid metabolism.

The hormone GLP-1, secreted by intestinal cells, promotes insulin secretion and inhibits glucagon release. Studies have shown that GLP-1 plays a crucial role in ameliorating metabolic disorders and obesity-related diseases. This mechanism suggests that *E. faecalis* may improve hepatic metabolism and inflammatory status through a similar pathway, thereby reducing liver weight. Furthermore, Zhang et al. [29] demonstrated that the addition of mixed probiotics increased GLP-1 levels in hens, which is consistent with the findings of this study. Leptin regulates neural structures by modulating neurons expressing brain-derived neurotrophic factor (BDNF) in the hypothalamic paraventricular nucleus, thereby promoting fat breakdown and utilization. In the present study, serum leptin levels were significantly elevated on day 25 of the experiment, aligning with the expected results. Additionally, previous studies have shown that probiotic supplementation significantly increases serum levels of GLP-1, insulin, and glucose in aged laying hens [30], which is largely consistent with the results of this study. However, no significant changes in glucagon levels were observed in this trial, possibly due to species-specific differences.

The present study revealed that supplementation with *E. faecalis* exerts time-dependent and complex effects on hepatic lipid metabolism. Previous studies have demonstrated that the regulatory effects of probiotics on lipid metabolism are time-dependent: in the short term, they reduce TG levels by promoting fatty acid oxidation, whereas long term supplementation may activate lipid synthesis pathways, leading to elevated TG levels [31]. Additionally, probiotics have been shown to significantly reduce FFA levels, but their effects on TG levels exhibit a time-dependent pattern, with a reduction in the short term and a potential increase over the long term [32], which is consistent with the findings of this study. Notably, during the later stages of the experiment, the increased energy demands of the animals likely triggered enhanced hepatic TG synthesis to meet energy storage requirements [33]. This may explain the observed significant decrease in serum TG levels alongside a significant increase in hepatic TG levels on day 45.

The liver is a central organ in lipid metabolism, and an imbalance between lipid synthesis and catabolism can lead to excessive fat deposition in the liver [34]. *ACCA* and *FASN* are rate-limiting enzymes in de novo fatty acid synthesis [35] and key determinants of lipogenic capacity. In the present study, the mRNA expression levels of *FASN* in the *E. faecalis* group were significantly higher than those in the control (CON) group on days 25 and 45, suggesting that *E. faecalis* supplementation may enhance lipogenic capacity in geese. Insulin is known to induce *FASN* promoter activity, thereby promoting its expression [36]. However, the significant increase in *FASN* mRNA levels may also indicate that *E. faecalis* enhances fatty acid oxidation to a greater extent than fatty acid synthesis, leading to a reduction in hepatic FFA levels. *FXR*, a bile acid receptor [37,38], plays a critical role in the regulation of lipid metabolism. Hepatic steatosis is often accompanied by the downregulation of *FXR* expression [39], and the disruption of the *FXR* gene results in fat accumulation in the blood and liver [40]. In contrast, *FXR* activation promotes fatty acid oxidation and reduces hepatic fat deposition by upregulating *PPARα* gene expression [41]. In this study, the significant increase in *FXR* and *PPARα* mRNA levels on day 25 suggests that *E. faecalis* may enhance fatty acid oxidation by activating the *FXR-PPARα* signaling pathway. *PPARα*, a ligand-activated transcription factor, directly or indirectly regulates hepatic lipogenic pathways [42]. The significant upregulation of *PPARα* expression on day 25 aligns with the expected results, further supporting the time-dependent regulation of fatty acid oxidation by *E. faecalis*. However, we observed opposing trends in the expression levels of *PPARα* on days 25 and 45 of the experiment. This phenomenon may be attributed to the adaptive responses of the gut microbiota and host metabolism to *E. faecalis* supplementation. These dynamic changes reflect the complex regulatory effects of *E. faecalis* on lipid metabolism, which may involve time-dependent modulation of metabolic pathways. This observation provides valuable insights for future research, and we plan to further investigate the underlying mechanisms. *SREBP-1*, a key lipogenic transcription factor, plays an essential role in maintaining cholesterol homeostasis [43]. On day 5 of the experiment, the mRNA expression of *SREBP-1* was significantly reduced, as is consistent with the expected results, indicating that *E. faecalis* may promote fatty acid oxidation by suppressing lipid synthesis. However, on days 25 and 45, *SREBP-1* expression was significantly increased, which contrasts with the expected outcomes. This phenomenon may be attributed to increased energy demands and adaptive changes in gut microbiota during the later stages of the experiment, leading to the activation of lipogenic pathways to meet energy storage requirements. These findings highlight the complex time-dependent regulatory effects of *E. faecalis* on lipid metabolism.

The morphometric parameters of VH, CD, and the VH/CD ratio are common indicators of intestinal mucosal barrier function and gut health [44]. Previous studies have demonstrated that increased villus height and an elevated VH/CD ratio can enhance body weight and significantly improve growth performance [45]. In the present study, the addition of *E. faecalis* to drinking water increased the villus length in the ileum of the *E. faecalis* group. Similarly, the VH/CD ratio was also significantly improved. These results align with expectations, indicating that *E. faecalis* promotes ileal morphological development and enhances nutrient digestion and absorption. Furthermore, on day 5 of the experiment, the crypt depth in the ileum was significantly increased, while the VH/CD ratio was significantly reduced, which may partially explain the notable decrease in body weight observed during the first 5 days of the trial.

Oil Red O staining is a widely used histochemical method for detecting neutral lipid deposition in tissues or cells [46]. In the present study, on day 25 of the experiment, the *E. faecalis* group exhibited significantly lower hepatic lipid deposition compared to the CON group, indicating that *E. faecalis* supplementation effectively reduces lipid accumulation in the liver. Previous studies have shown that heat-inactivated Lactobacillus reuteri GMNL-263 significantly decreases hepatic lipid accumulation in rats [47], which is consistent with the findings of this study. These results align with expectations and are supported by the regulatory effects of *E. faecalis* on lipid metabolism-related genes, suggesting that *E. faecalis* may improve hepatic lipid metabolism by promoting fatty acid oxidation and inhibiting lipid synthesis. Furthermore, *E. faecalis* may indirectly influence hepatic lipid metabolism by modulating the gut microbiota and its metabolites, such as short-chain fatty acids, thereby further reducing lipid deposition.

The diversity and stability of the gut microbial community play a crucial role in promoting gut barrier function and enhancing the immune system. Notably, gut microbial diversity is significantly reduced in obese individuals [48], and strong links between the gut microbiota and adipose tissue were established [49]. Existing studies suggest that probiotics may mitigate fat accumulation and low-grade inflammation in metabolic tissues by modulating the gut microbiota [50]. In this study, after a comprehensive analysis of various pre-serum and hepatic indices, the ileal contents of geese on day 25 were examined to investigate changes in the bacterial flora. On day 25 of the experiment, the *E. faecalis* group exhibited a higher abundance and greater diversity of bacterial species in the ileum. These findings suggest that *E. faecalis* may enhance slaughter performance by altering the richness and composition of the ileal gut microbiota in geese.

Regarding microbial species composition, the *E. faecalis* group exhibited significant differences compared to the CON group in the relative abundances of *Corynebacteriaceae*, *Bacillus*, *Bacillus marisflavi*, *Micromonosporaceae*, and *SMB53*. *Bacillus* species are known to promote lipid metabolism in the host [51]. For instance, supplementation with Bacillus subtilis SPB1 lipopeptide biosurfactant was shown to reduce renal fat deposition in rats fed a high-calorie diet, attributed to its hypoglycemic and antihypertensive effects [52]. Similarly, *Bacillus licheniformis* was reported to reduce body weight and improve glucose tolerance, obesity, and insulin resistance in high-fat diet-fed mice by modulating the composition of the colonic microbiota [53]. Additionally, *Micromonosporaceae* are recognized for their antimicrobial properties, as exemplified by the gentamicin C complex produced by *Micromonospora spinosa*, which is a globally significant antibiotic [54].

Correlation analysis revealed a significant positive relationship between Actinobacteria and lipid metabolism indices. Certain strains of *Actinobacteria* are capable of utilizing fatty acids and oils as carbon sources, catabolizing and incorporating these compounds into lipid metabolic pathways. In contrast, *SMB53*, *Solibacillus*, and *Corynebacterium* exhibited significant negative correlations with lipid metabolism indicators. Notably, *SMB53* was reported to be increased in obesity-susceptible mice [55]. *Solibacillus* was proposed as a key microbe associated with inflammation and lipid metabolism [56]. Furthermore, a combination of fungal polysaccharides, ginkgo polysaccharides, and hawthorn flavonoids was shown to reduce the relative abundance of *Corynebacterium-1* in the intestinal microbiota of high-fat diet-fed rats [57]. The observed improvement in lipid metabolism may be attributed to the significant increase in *Actinobacteria* abundance and the notable decreases in the abundances of *SMB53*, *Solibacillus*, and *Corynebacterium*.

## 5. Conclusions

Drinking water containing *E. faecalis* at a concentration of 1.0 × 10^8^ CFU/mL promoted the oxidative breakdown of fatty acids in geese, reduced blood lipids and hepatic lipid deposition, and improved the ileal intestinal morphology and ileal intestinal flora. These effects improved growth performance and lipid metabolism in geese.

## Figures and Tables

**Table 1:** Nutrient content and composition of basal diet (air-dry basis) %.

Ingredients	Content	Nutrient Levels	Content
Corn	61.50	ME/(MJ/Kg) ^2^	10.92
Soybean meal	25.00	Crude protein	16.02
Rice bran	7.00	Crude fiber	5.60
Wheat bran	3.00	Calcium	0.72
NaCl	0.30	Phosphorus	0.54
Vitamin and mineral premix ^1^	1.00	Lysine	0.80
DL-Met	0.10	Methionine	0.33
Dicalcium phosphate	1.10		
Mountain flour	1.00		

^1^ The vitamin and mineral premix offers each kilogram of concentrate: Zn 60 mg, Cu 50 mg, Mn 80 mg, Fe 50 mg, I 1.6 mg, Se 12 mg, vitamin A 30,000 IU, vitamin E 100 mg, and vitamin D3 100,000 IU. ^2^ ME was a computed value, while measurements were used to quantify other quantities.

**Table 2:** Primers of RT-qPCR.

Gene Name	Primer Sequence (5′–3′)
*ACCA*	F: CCGGGAGGTTAATGGAAGGAC
	R: TGTGCCCTCAGCACTCTTG
*SREBP-1*	F: CCGCTCATCCATCAACGAC
	R: GGCTGAGGTTCTCCTGCTTC
*FXR*	F: TTTGCTCCAGCTGGACTCAG
	R: AGAAAGAGACGGTAGTTCCAGAG
*FASN*	F: GCCTGCCACAACTCTGAAGATAC
	R: CTCCTTTGCGAACACACCATCC
*PPARα*	F: CCACAGCTCCAGGTAGCATAG
	R: AGGCACTTTTGAAAACGACAG
*β-actin*	F: CCCAGCCATGTATGTAGCCATCC
	R:AACACCATCACCAGAGTCCATCAC

F: Forward primer; R: reversed primer. Abbreviations: *ACCA* = acetyl-CoA carboxylase; *SREBP-1* = sterol regulatory element-binding protein 1; *FXR* = farnesoid X receptor; *FASN* = fatty acid synthase; *PPARα* = peroxisome proliferator-activated receptor α. Note: β-actin (mRNA and lncRNA) were selected as reference genes.

**Table 3:** The effect of drinking water with a concentration of 1.0 × 10^8^ CFU/mL *Enterococcus faecalis* on geese growing performance.

Item	Groups ^1^	
	CON	*E. faecalis*
BW (g)		
1 d	350.00 ± 9.79	346.00 ± 9.79
5 d	528.00 ± 24.45 ^a^	468.00 ± 24.45 ^b^
25 d	1380.00 ± 28.35 ^b^	1742.00 ± 28.35 ^a^
45 d	2150.00 ± 97.54 ^b^	2592.00 ± 97.54 ^a^
ADG (g/d)		
1 to 5 d	35.60 ± 3.57 ^a^	24.40 ± 3.57 ^b^
6 to 25 d	42.60 ± 0.92 ^b^	63.70 ± 0.92 ^a^
26 to 45 d	38.50 ± 4.07	42.50 ± 4.07
1 to 45 d	40.00 ± 2.01 ^b^	49.91 ± 2.01 ^a^
ADFI (g/d)		
1 to 5 d	233.80 ± 2.97	229.60 ± 2.97
6 to 25 d	412.00 ± 2.58 ^a^	330.20 ± 2.58 ^b^
26 to 45 d	590.80 ± 2.66 ^a^	526.80 ± 2.66 ^b^
1 to 45 d	471.67 ± 1.86 ^a^	406.40 ± 1.86 ^b^
FCR		
1 to 45 d	11.80 ± 0.36 ^a^	8.19 ± 0.36 ^b^

Abbreviations: BW = body weight; ADG = average daily gain; ADFI = average daily feed intake; FCR = feed conversion ratio; ^1^ CON = control group; *E. faecalis* = concentration of *Enterococcus faecalis* was 1.0 × 10^8^ CFU/mL group. ^a,b^ Means within row with different letters are statistically significant (*p* < 0.05). Experimental results are expressed as mean ± standard error of mean (SEM).

**Table 4:** The impact of consuming water containing 1.0 × 10^8^ CFU/mL *Enterococcus faecalis* on the slaughter metrics of geese.

Item	Groups ^1^	
	CON	*E. faecalis*
Abdominal Fat (g)		
5d	28.00 ± 1.20 ^a^	10.00 ± 1.20 ^b^
25d	18.80 ± 2.84	21.20 ± 2.84
45d	130.40 ± 7.38 ^a^	86.80 ± 7.38 ^b^
Liver weight (g)		
5d	23.42 ± 1.83 ^a^	18.76 ± 1.83 ^b^
25d	49.30 ± 3.86	57.16 ± 3.86
45d	91.00 ± 3.76 ^a^	60.92 ± 3.76 ^b^
Half-eviscerated weight (g)		
5d	397.06 ± 18.39 ^a^	351.94 ± 18.39 ^b^
25d	1037.76 ± 21.32 ^b^	1309.98 ± 21.32 ^a^
45d	1616.80 ± 73.35 ^b^	1949.18 ± 73.35 ^a^

^1^ CON = control group; *E. faecalis* = concentration of *Enterococcus faecalis* was 1.0 × 10^8^ CFU/mL group. ^a,b^ Means within row with different letters are statistically significant (*p* < 0.05). Experimental results are expressed as mean ± standard error of mean (SEM).

## Data Availability

The data presented in this study are available on request from the corresponding author.
